# Membrane thinning and lateral gating are consistent features of BamA across multiple species

**DOI:** 10.1371/journal.pcbi.1008355

**Published:** 2020-10-28

**Authors:** Jinchan Liu, James C. Gumbart

**Affiliations:** 1 School of Physics, Georgia Institute of Technology, Atlanta, Georgia, United States of America; 2 Tang Aoqing Honors Program in Science, College of Chemistry, Jilin University, Changchun, Jilin Province, China; University of Kansas, UNITED STATES

## Abstract

In Gram-negative bacteria, the folding and insertion of β-barrel outer membrane proteins (OMPs) to the outer membrane are mediated by the β-barrel assembly machinery (BAM) complex. Two leading models of this process have been put forth: the hybrid barrel model, which claims that a lateral gate in BamA’s β-barrel can serve as a template for incoming OMPs, and the passive model, which claims that a thinned membrane near the lateral gate of BamA accelerates spontaneous OMP insertion. To examine the key elements of these two models, we have carried out 45.5 μs of equilibrium molecular dynamics simulations of BamA with and without POTRA domains from *Escherichia coli*, *Salmonella enterica*, *Haemophilus ducreyi* and *Neisseria gonorrhoeae*, together with BamA’s homolog, TamA from *E. coli*, in their native, species-specific outer membranes. In these equilibrium simulations, we consistently observe membrane thinning near the lateral gate for all proteins. We also see occasional spontaneous lateral gate opening and sliding of the β-strands at the gate interface for *N. gonorrhoeae*, indicating that the gate is dynamic. An additional 14 μs of free-energy calculations shows that the energy necessary to open the lateral gate in BamA/TamA varies by species, but is always lower than the Omp85 homolog, FhaC. Our combined results suggest OMP insertion utilizes aspects of both the hybrid barrel and passive models.

## Author summary

Gram-negative bacteria such as *Escherichia coli* have a second, outer membrane surrounding them. This outer membrane provides an additional layer of protection, but also presents an additional challenge in its construction, exacerbated by the lack of chemical energy in this region of the bacterial cell. For example, proteins in the outer membrane are inserted via BamA, itself an integral membrane protein. The precise mechanisms by which BamA assists in the insertion process are still unclear. Here, we use extensive simulations in atomistic detail of BamA from multiple species in its native outer membrane environment to shed light on this process. We find that the lateral gate of BamA, a proposed pathway into the membrane, is dynamic, although to a degree varying by species. On the other hand, thinning of the outer membrane near BamA’s lateral gate is observed consistently across all simulations. We conclude that multiple features of BamA contribute to protein insertion into the outer membrane.

## Introduction

Gram-negative bacteria are enveloped by two membranes, an inner membrane and an outer membrane (OM) [1]. This OM serves as a strong barrier against drugs targeting Gram-negative bacteria [2] with lipopolysaccharides/lipooligosaccharides (LPS/LOS) on the outer leaflet and a mixture of phospholipids on the inner leaflet [3, 4]. The outer membrane proteins (OMPs) in the asymmetric OM play important roles in nutrient transport [5, 6], waste export [7, 8], cell signaling [9, 10] and membrane biogenesis [11, 12] and are almost exclusively β-barrel structures [13]. The folding and insertion of these β-barrel OMPs are mediated by the β-barrel assembly machinery (BAM) complex without ATP or ion gradients [14–16]. Although multiple structures have been solved by X-ray crystallography or cryo-electron microscopy, the exact mechanisms of the process remain unknown [17–20]. In *E. coli*, the BAM complex consists of five members, named BamA-E [16, 21]. BamA contains a 16-strand transmembrane β-barrel and five periplasmic polypeptide-transport-associated (POTRA) domains [22]. BamB-E, which interact with BamA primarily through its POTRA domains, are lipoproteins with their N-termini embedded in the OM [23–25]. Experiments have shown that although BamA itself is able to accelerate OMP folding in vitro [26], BamD is also required for cell viability in vivo [27], and all BAM components are required to achieve maximum efficiency [28, 29].

**Fig 1:**
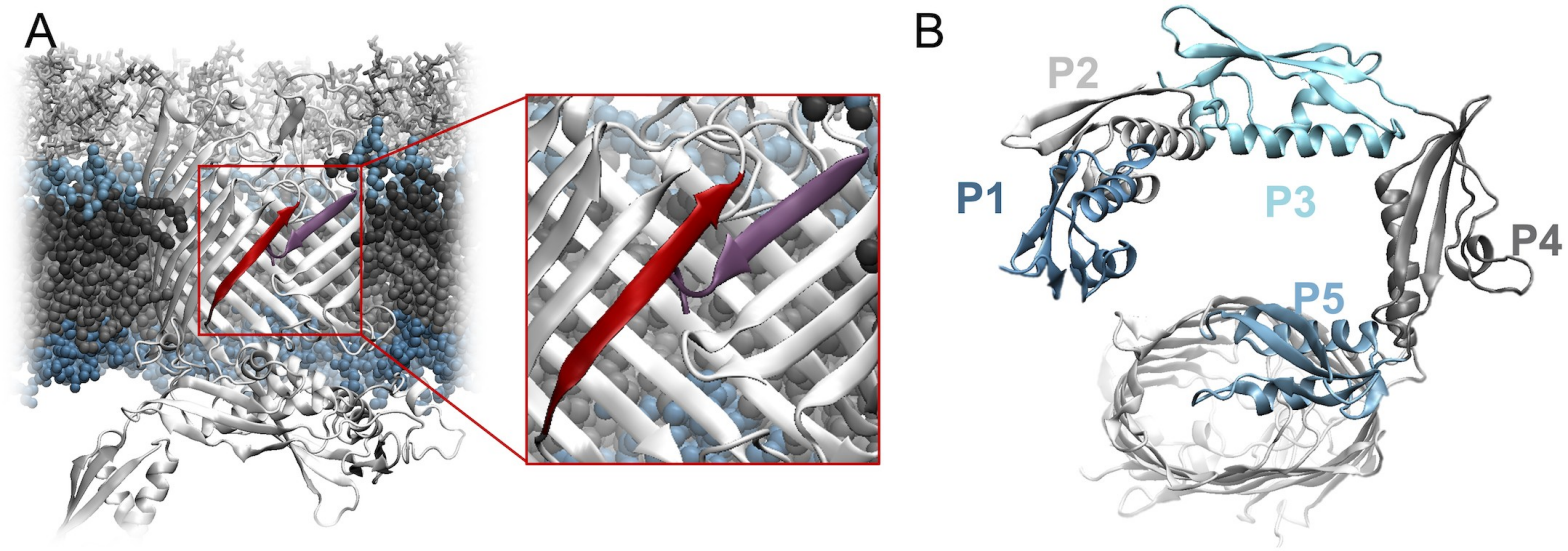
BamA structure. (A) BamA of *N. gonorrhoeae* in its OM with protein in white. β1 is highlighted in red and β16 is highlighted in purple. (B) Five POTRA domains of BamA of *N. gonorrhoeae* in tandem with different colors.

The central component of the BAM complex, BamA, is a member of the Omp85 family of proteins that is essential for OM biogenesis in mitochondria, chloroplasts and bacteria [30, 31]. Several structural features of BamA have been recognized and highlighted, leading to two corresponding mechanistic models of insertion [32]. The seam of the BamA barrel between β1 and β16 strand (Fig 1A) is dynamic [33], and it can open laterally [22, 34]. This opening may serve as a template for nascent proteins to initiate β-barrel formation through β-augmentation, referred to as the hybrid barrel model [35]. The hybrid barrel model is supported by a series of experiments [36], including a recent structure of two BamAs, one joined to the other at their lateral gates [37]. Alternatively, the passive model focuses on the narrowed hydrophobic surface at the lateral gate, which is predicted to disorder the membrane and attenuate its thickness [22, 34]. The thinner, less stable membrane would allow OMPs to fold into it directly [38, 39]. Additionally, a homolog of BamA, TamA also consists of a 16-stranded β-barrel at its C-terminus and three POTRA domains at its N-terminus [40]. TamA’s POTRA3 is similar to BamA POTRA domains, and it has been suggested that the function of the β-barrels of BamA and TamA is conserved [41].

With the exact mechanisms of OMP insertion by BamA and TamA unknown, we investigated the structural features of BamAs of different species together with TamA of *E. coli* using molecular dynamics (MD) simulations with each embedded in its respective native OM. We constructed 12 systems with and without POTRA domains and ran 45.5 μs of equilibrium simulations as well as over 14 μs of replica-exchange umbrella sampling (REUS) to probe the lateral gating of BamA (see Table 1 for the full list). Our simulations show that the frequency of lateral gating for BamA is species dependent while membrane thinning for BamA is species independent, thus shedding light on potential insertion mechanisms.

**Table 1:** A summary of equilibrium simulations and REUS simulations performed for this study.

Label	Equilibrium Simulations		REUS Simulations	
	Time × Replica	Sum	Replica × Time	Sum
EcBamA	2000 ns × 2	4000 ns	18 × 70 ns	1260 ns
EcBamAΔP	2000 ns × 2	4000 ns	18 × 60 ns	1080 ns
EcBamAcw	1500 ns × 1	1500 ns		
SeBamAΔP	2000 ns × 2	4000 ns	17 × 50 ns	850 ns
HdBamA	2000 ns × 2	4000 ns	17 × 100 ns	1700 ns
HdBamAΔP	2000 ns × 2	4000 ns	17 × 65 ns	1105 ns
NgBamA	2000 ns × 2	4000 ns	17 × 100 ns	1700 ns
NgBamAΔP	2000 ns × 2	4000 ns	18 × 50 ns	900 ns
EcTamA	2000 ns × 2	4000 ns	16 × 90 ns	1440 ns
EcTamAΔP	2000 ns × 2	4000 ns	17 × 90 ns	1530 ns
BpFhaC	2000 ns × 2	4000 ns	19 × 70 ns	1330 ns
BpFhaCΔP	2000 ns × 2	4000 ns	20 × 75 ns	1500 ns

## Materials and methods

### System construction

We constructed 12 membrane-protein systems in total with and without POTRA domains (ΔP) for this study using the CHARMM-GUI membrane builder [42, 43]. All proteins were placed in asymmetric outer membranes (OM) consisting of a phospholipid (PL) inner leaflet and a lipopolysaccharide (LPS) outer leaflet with O-antigen excluded. The composition of the inner leaflet is decided by species. We assigned lipid tails with different degrees of unsaturation and different numbers of carbons to different types of phospholipid (phosphatidylethanolamine, phosphatidylglycerol, phosphatidylcholine and cardiolipin are considered in this study) to satisfy the composition of lipid tails as well as the composition of lipid types [44–49]. We modelled LPS with their respective lipid A and core oligosaccharides for *E. coli* [50], *S. enterica* [50], *H. ducreyi* [51, 52], *N. gonorrhoeae* [53] and *Bordetella cepacia* [54, 55]. All systems are solvated with TIP3P water [56]. Magnesium ions were used to neutralize the negative charges of LPS while 0.15 M KCl was used to neutralize the systems. See S1 Table for detailed information of all systems.

An additional system starting from EcBamA was also constructed. In this system, a model of the *E. coli* peptidoglycan (PG) cell wall [57, 58] was added in the periplasm. The PG was anchored to the OM via five Lpp trimers (PDB 1EQ7 [59]), which are tri-acylated at their N-termini (inserted into the inner leaflet of the OM) and covalently bonded to PG for one out of every three copies at their C-terminal end [60]. The number of Lpp was selected to be commensurate with the roughly 500,000 copies per cell [1].

### MD Protocol

We ran simulations in both NAMD [61] and Amber [62]. We used the CHARMM36m force field for proteins [63] and CHARMM36 for lipids [64]. Langevin dynamics (damping constant *γ* = 1.0 ps^−1^) was used to keep the temperature (310 K) constant, and an anisotropic Langevin piston barostat (in NAMD) or a Monte Carlo barostat (in Amber) was used to enforce constant pressure (1 atm) [65]. The time step of all simulations without hydrogen mass repartitioning (HMR) was 2 fs. Bonded interactions and short-range nonbonded interactions (less than the 12-Å cutoff) are calculated at every time step. The Particle-mesh Ewald (PME) method was used for long-rang interactions, updated every other time step [66]. VMD was used to analyze all results [67].

#### Equilibrium Simulations

All systems were equilibrated first in NAMD by releasing system components sequentially (lipid tails for 1 ns, everything except protein for 10 ns, everything except protein backbone for 10 ns, everything for 10 ns). The systems were minimized for 2000 steps before each step. After equilibration, all systems but the PG-containing one were run twice in Amber for 2000 ns each using HMR and a time step of 4 fs [68, 69]. Because systems with bonds crossing periodic boundaries cannot be run in Amber, the PG-containing system was run in NAMD for 1500 ns using HMR.

#### Replica Exchange Umbrella Sampling (REUS) simulations

We used REUS to calculate the potential of mean force (PMF) for lateral gate opening [70]. Targeted Molecular Dynamics (TMD) was used to generate starting states for REUS [71] (see S1 Fig for more details about TMD). The collective variable for BamA of *N. gonorrhoeae* is defined as the distance between N and O atoms that potentially form hydrogen bonds at the lateral gate projected to the direction that lateral gates open (see S2 Fig). The colvars module of NAMD was used to construct all collective variables [72]. A total of 17-20 unevenly distributed windows were used for REUS, covering a range from 2-12 Å. Different force constants were used for different windows during REUS: see S2 Table for centers and force constants. The sampling data of REUS was used to calculate the PMF using the weighted histogram analysis method (WHAM) [73]. Each REUS simulation was run until an additional 5 ns changed the PMF endpoint by less than 0.2 kcal/mol at which point it was considered to be converged (see S3 Fig).

## Results

### Membrane hydrophobic thickness of BamA and TamA systems

**Fig 2:**
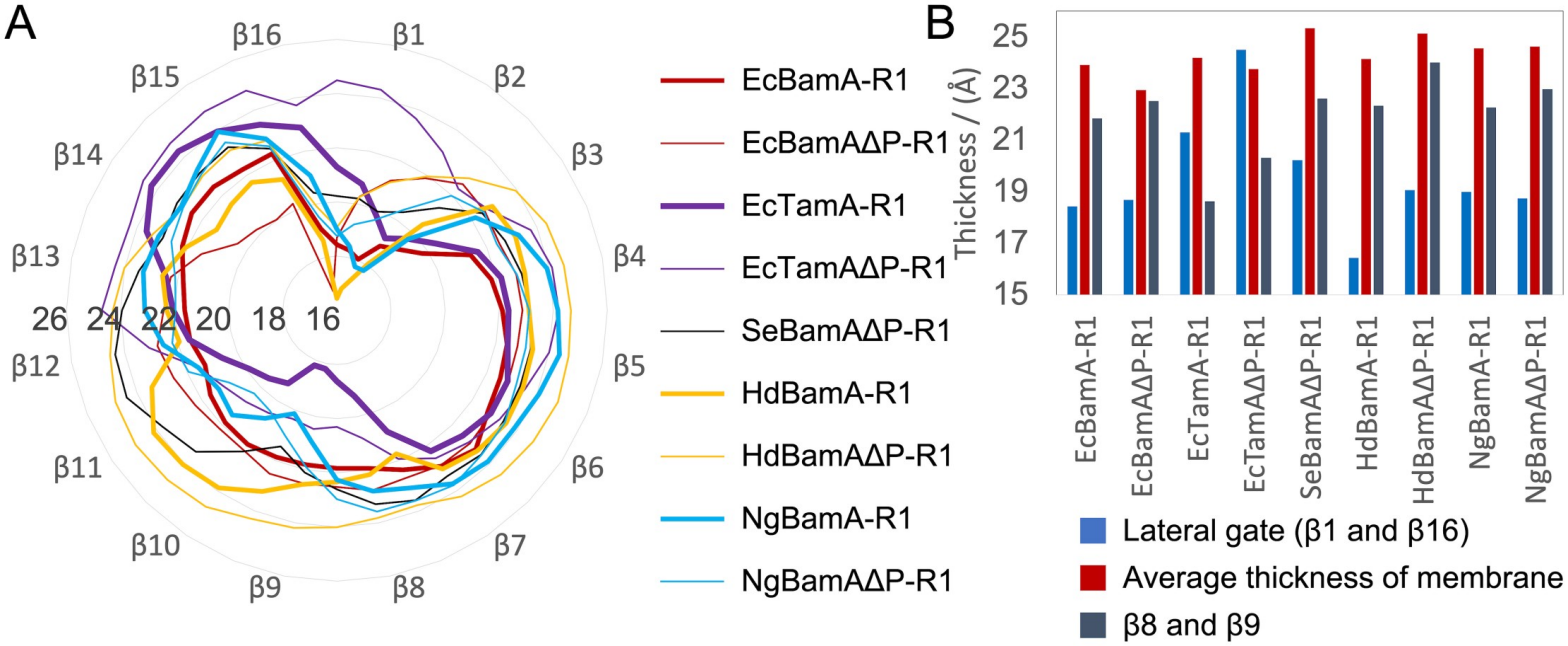
Membrane thickness in equilibrium simulations. (A) Average thickness around the β-barrel for the first run of each system (See S6 Fig for all systems). Labeled concentric circles represent the membrane thickness in Å. (B) Average thickness of the whole membrane (in red) as well as average thickness near β8 and β9 at the back of the β-barrel in grey and near the lateral gate in blue.

BamAs exhibit narrowed hydrophobic surfaces at their lateral gates, which is predicted to thin the membrane and create a disordered lipid region [22]. Previous MD simulations of BamA of *E. coli*, *H. ducreyi* and *N. gonorrhoeae* supported this membrane thinning [22, 34], which can accelerate nascent proteins folding and inserting into the OM [39, 74]. In our 2-μs equilibrium simulations, we observed membrane thinning near different strands of the barrel in different systems (Fig 2A). For BamA of *E. coli*, *S. enterica*, *H. ducreyi* and *N. gonorrhoeae*, membranes near the lateral gate are thinned by 5.3±1.7 Å, demonstrating that the membrane thinning at the lateral gate is a universal feature of BamA (Fig 2B and S4 Fig). We also quantified the extent of membrane thinning by measuring the area of the membrane for which the thickness is below 20 Å (S5 Fig). The results indicate that the areas can be very different, ranging from 4 Å^2^ (in one replica of HdBamAΔP) to 768 Å^2^ (in one replica of EcBamAΔP), with the average at ∼350 Å^2^. This average area is slightly more than the cross-sectional area of an eight-stranded *β*-barrel, suggesting that it is of sufficient extent to accelerate passive, concerted insertion of at least small OMPs.

Some systems also exhibit minor thinning near β8-β10 strands (Fig 2B). Although TamA’s structure is broadly similar to BamA’s, TamA systems have the most obviously thinned membrane near the β8-β10 strands (See S6 Fig for the average thickness near each strand for all replicas). For EcTamA, membranes near β8 and β9 are thinned by 3.7±1.7 Å. When we inspect the structure of TamA, we find a series of charged residues, R394, R396, R398, D407 and R437 on the periplasmic side of β8, β9 and β10 resulting in membrane thinning. Additionally, three hydrophilic residues, Q409, Y411 and N433, in the middle of β8, β9 and β10 contribute to the membrane defect (S7 Fig) [75].

### Spontaneous lateral gate opening and sliding

**Fig 3:**
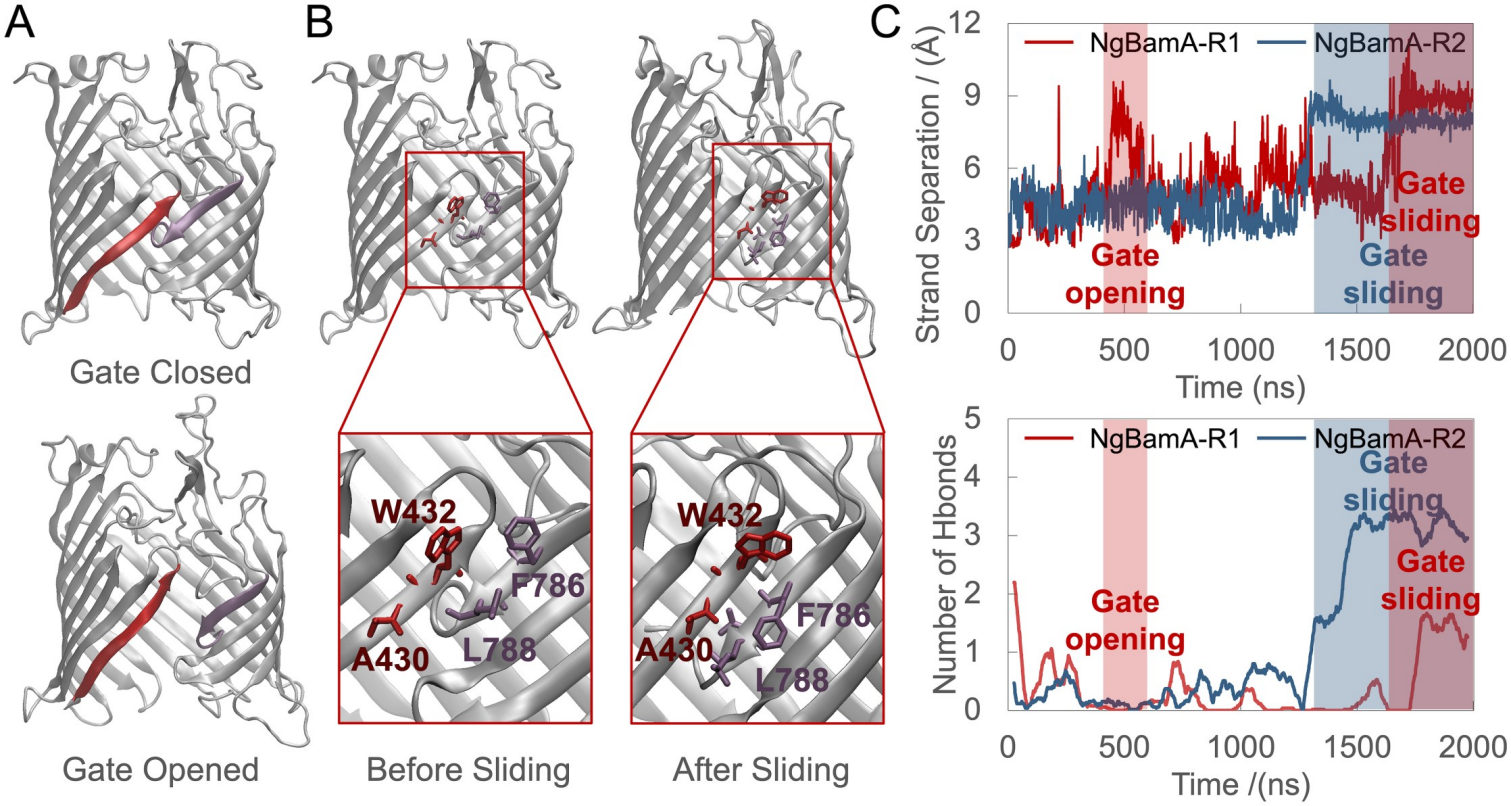
Lateral gate opening and sliding in equilibrium simulations. (A) Snapshots of a closed state of NgBamA (top) and the maximum strand separation in NgBamA (bottom) with β1 in red and β16 in purple. (B) Snapshots of β-barrel of NgBamA before (left) and after (right) the sliding. (C) Strand separation (top) and number of hydrogen bonds between backbones of β1 and β16 (bottom) of NgBamA system over time. Strand separation is defined by the average of the distance between O of W432 and N of L788 and the distance between N of W432 and O of L788 in NgBamA. A cutoff distance of 3.5 Å and an angle of 30 degrees are used to define a hydrogen bond.

Previously, spontaneous lateral gate opening of the barrel of *N. gonorrhoeae* BamA in MD simulations was reported to take place in DMPE at 310 K [22], in DLPC at 310 K and 340 K, and in OM at 340 K [34]. Here we observed lateral gate opening of NgBamA in its native OM at 310K in our equilibrium simulations (Fig 3A). The β1 and β16 strands separate spontaneously with the number of hydrogen bonds formed between β1 and β16 dropping at the beginning of both replicas (Fig 3C). We also observed fluctuations of the lateral gates in HdBamAΔP, HdBamA, and NgBamAΔP systems (S8 Fig). Strand separation stays at 3 Å in SeBamAΔP, EcBamAΔP and EcBamA systems (S8 Fig), which matches the length of a hydrogen bond. TamA’s lateral gate also opens in equilibrium simulations, and it stays open due to a lipid that enters it (S8 Fig).

One of the most unexpected observations from our equilibrium simulations is sliding of the lateral gate (Fig 3B). In our NgBamA equilibrium simulation, W432 and L788 form hydrogen bonds initially. As the lateral gate opens, the hydrogen bonds break and the distance between W432 and L788 fluctuates. Then the distance sharply increases and stays above 8 Å while the number of hydrogen bonds rises simultaneously (Fig 3C), which results from F786 sliding down and forming hydrogen bonds with W432. Additionally, we observed sliding of the lateral gate in HdBamAΔP, which explains the apparent sharp increase in strand separation (S8 Fig). This observed sliding agrees with experimental results in which EcBamA can form disulfide cross-links between lateral gate residues (G433C-T809C and G431C-Q803C) that are 14 Å apart in the crystal structure [33], demonstrating its flexibility.

### Energetics of lateral gate opening

To further investigate lateral gate opening, we ran replica-exchange umbrella sampling (REUS) simulations to calculate the potential of mean force (PMF) for opening of all systems with and without POTRA domains. In order to avoid conflating the sliding conformation with the lateral gate opening conformation, we projected the distance of atoms forming hydrogen bonds at the lateral gate on the direction it opens. For example, we projected the vector that the N/O atoms of W432 and O/N atoms of L788 form in NgBamA (and the corresponding residues for other proteins) on their direction at maximum separation, defined as the lateral gate opening coordinate (S2 Fig).

We use FhaC as a control since it is also a member of the Omp85 family but does not assist with OMP insertion [76]. As expected, with or without POTRA domains, the PMFs of BamA and TamA are lower than those of FhaC [34]. Unexpectedly, although all PMFs tend to increase as the strands separate, a well in the PMF appears from 3.5 Å to 5.5 Å. This small drop in the PMFs is due to water molecules going between β1 and β16 and forming hydrogen bonds with the backbone, stabilizing the open state slightly (S9 Fig).

**Fig 4:**
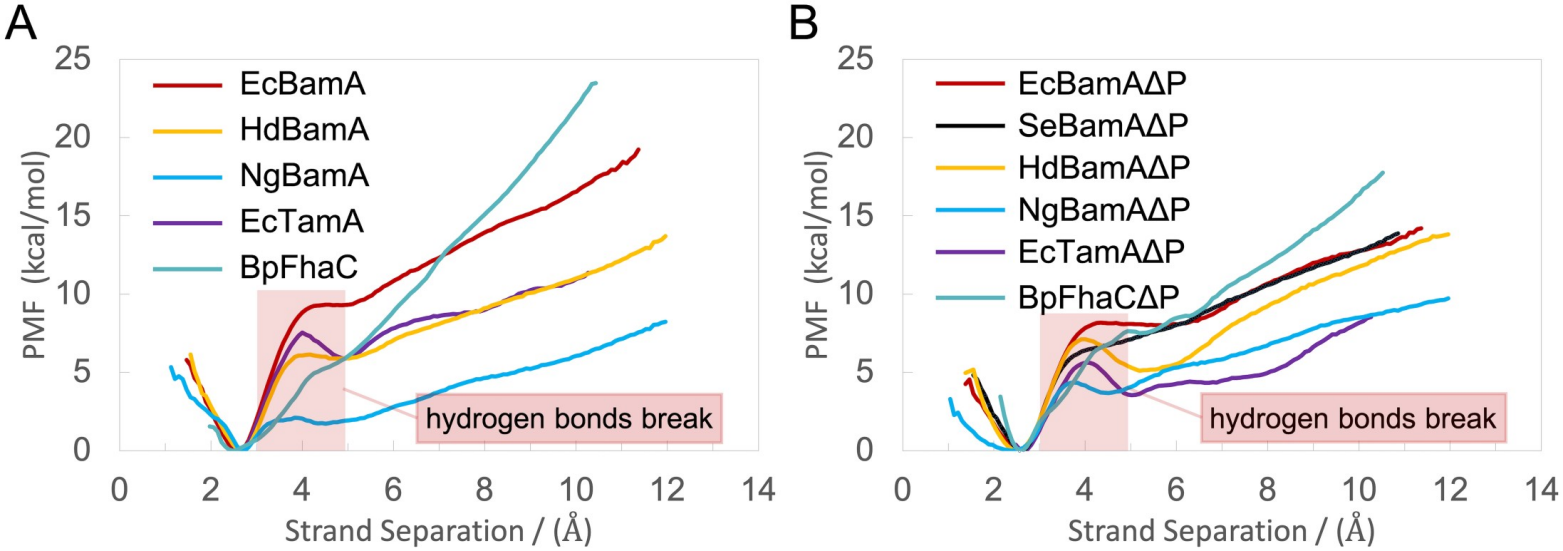
PMFs for lateral gate opening. (A) PMFs of lateral gate opening for systems with POTRA domains. (B) PMFs of lateral gate opening for barrel-only systems. In both panels, the region over which hydrogen bonds between the N- and C-terminal strands are ruptured is indicated.

The energy required to open the lateral gate for NgBamAΔP and EcTamAΔP is about 4 kcal/mol lower than that of EcBamAΔP, SeBamAΔP and HdBamAΔP (Fig 4B). The PMFs for systems with POTRA domains are within a relatively larger range from 8 to 20 kcal/mol compared to barrel-only systems (Fig 4A). Interestingly, the PMF of lateral gate opening for NgBamA is sufficiently low to happen spontaneously on the microsecond timescale [34], as observed in our equilibrium simulations. Opposite from NgBamA, whose PMF is lower than NgBamAΔP’s, the PMFs for EcBamA and EcTamA are both higher than those of their respective barrel-only systems. Finally, the PMFs for HdBamA and HdBamAΔP are similar to each other.

### Interactions between β-barrels and their environments

Based on our equilibrium simulations, we quantified the interaction propensity of each β-barrel residue with lipids, LPS, water, and other protein residues. For each residue, the number of atoms within 4 Å of its side chain for each type of molecule is accumulated over the two 2-μs runs. We plot the percentage for each type in S10 Fig. Unsurprisingly, the 16 β-stands primarily interact with lipids while loops and turns interact with water, as previously reported for another OMP, OmpLA [77]. Additionally, it is evident that the β-strand residues whose side chains are inside the barrel are interacting more with water, while the β-strand residues whose side chains are outside the barrel are interacting with lipid A head group, lipid A tail, PL tail and PL head group periodically.

We developed a metric to quantify the overall difference in the interaction propensities between pairs of proteins (Table 2 and S11 Fig). Looking at EcBamA, for example, we find that SeBamA has the most similar interaction pattern, followed by (in order) HdBamA, NgBamA, EcTamA, and BpFhaC. These differences are consistent with the evolutionary relationships between these bacteria and/or proteins [78], further demonstrating that evolution operates within constraints presented by each protein’s local environment.

**Table 2:** Measure of the difference of interaction propensities between the proteins. Briefly, this difference is a Euclidean distance between each set of interaction propensities, averaged over all *aligned* residues between two proteins. See S11 Fig for further details of the calculation.

	EcBamA	SeBamAΔP	HdBamA	NgBamA	EcTamA	BpFhaC
EcBamA	0	0.079	0.120	0.156	0.172	0.218
SeBamAΔP	0.079	0	0.118	0.149	0.176	0.221
HdBamA	0.120	0.118	0	0.152	0.184	0.226
NgBamA	0.156	0.149	0.152	0	0.178	0.234
EcTamA	0.172	0.176	0.184	0.178	0	0.219
BpFhaC	0.218	0.221	0.226	0.234	0.219	0

One of the most prominent features of the interaction pattern is that some loop residues interact predominantly with water, making them attractive targets of novel antibiotics [79–81]. To better quantify their exposure, we calculated the ratio of each loop residue’s contact area with water to its surface area (S12 Fig). A series of residues on L4, L6 and L7 of EcBamA and SeBamAΔP, which are on the surface of the barrel lid, stand out. The results are consistent with the recent finding that N534, L536, V543, E554, Q561, D562, D568, D569, K644, E645 and L699 of EcBamA interact with a new peptide antibiotic [81]. Notably, loop residues of *E. coli* and *S. enterica* could be buried by O-antigen of the bacteria, which is not included in our *E. coli* and *S. enterica* systems [82]. In contrast, since the LPS in *N. gonorrhoeae* and *H. ducreyi* is a lower-weight form lipooligosaccharides (LOS), which lacks O-antigen [83], the residues on L4 and L7 of NgBamA as well as on L4 and L6 of HdBamA would always be exposed to water.

### POTRA domains interact with the OM and with Lpp

We observed extensive interactions between the POTRA domains of all BamAs and TamA with periplasmic leaflet of the OM, as was previously reported for EcBamA [84]. As the contact areas with the OM surface indicate (S13 Fig), the POTRA domains have a strong tendency to touch the membrane. Interestingly, the P1-P3 domains of TamA present levels of contact area similar to the corresponding POTRA domains of BamA of *E. coli*. For example, P2 of TamA has almost no contact with the OM, similar to P4 of EcBamA. All other POTRA domains of TamA and of EcBamA form extensive interactions with the periplasmic leaflet of the OM; their average contact areas over time ranges from 100 to 2000 Å^2^, which is considerable compared to the ∼5785 Å^2^ surface area on average for a POTRA domain (S3 Table).

**Fig 5:**
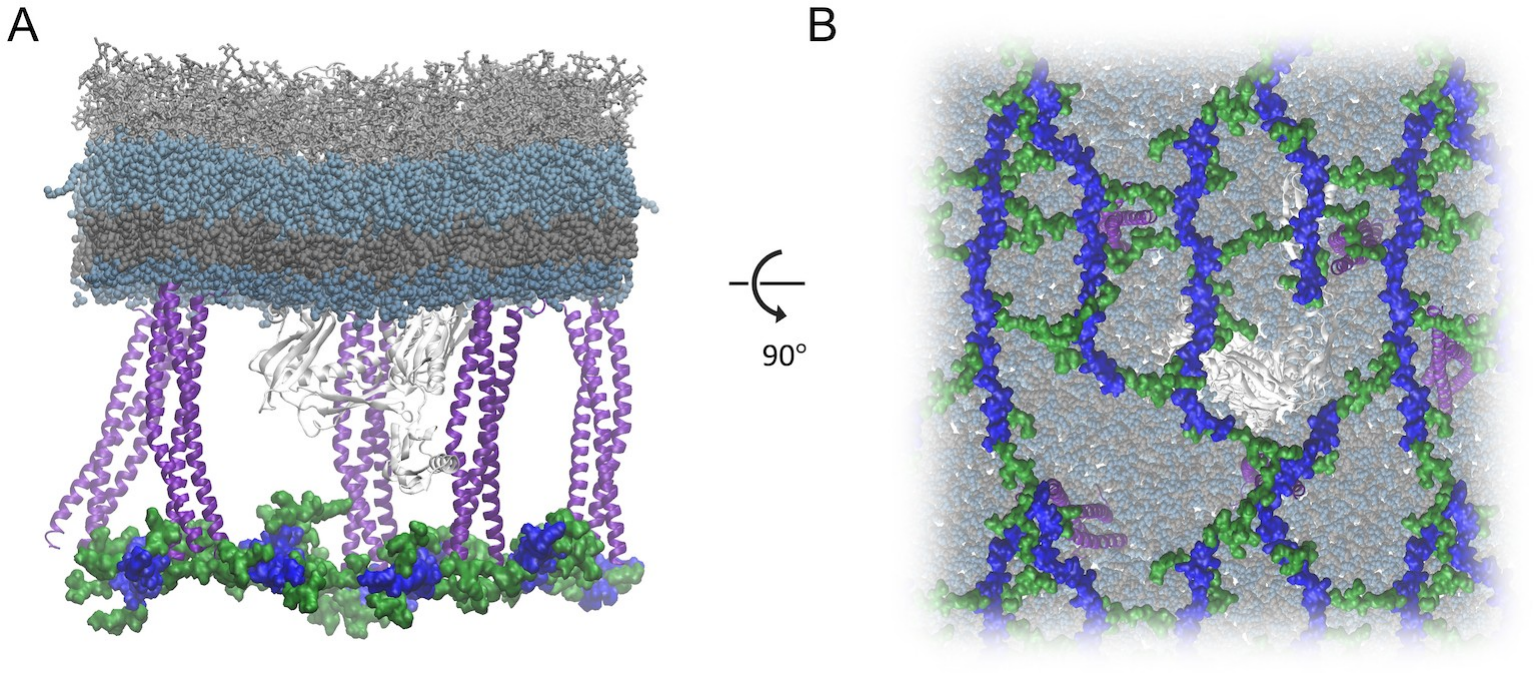
EcBamA with PG anchored by Lpp below. Glycan strands are in blue and peptides in green, viewed for the membrane plane (A) and periplasm (B). Lpp trimers are shown in purple; BamA is in white.

Peptidoglycan (PG) and its highly abundant protein anchor, Lpp, which positions the PG ∼8-10 nm from the bottom of the OM in the periplasm [57, 60], may affect the conformational dynamics of the POTRA domains. To examine this possibility, we ran a 1.5-μs simulation of EcBamA with PG anchored by Lpp below (Fig 5). Indeed, we observed a number of hydrogen bonds between the POTRA domains and Lpp (∼7 on average). Nonetheless, we still observed significant interactions between the POTRA domains and the OM as well (S13 Fig).

## Discussion

To interrogate the hybrid barrel model of OMP insertion and probe the dynamics of the lateral gate in BamA from different species, we ran a series of equilibrium simulations at 310 K with or without POTRA domains to examine the gate separation. In two 2-μs simulations for each of the nine BamA/TamA systems, we observed spontaneous lateral gate opening for *N. gonorrhoeae* BamA and *E. coli* TamA. No lateral gate opening is observed for *E. coli*, *S. enterica*, and *H. ducreyi* BamA, nor for our control protein, FhaC, on the 2-μs time scale. We next calculated the energetic landscape of lateral gate opening, which supports the observations from the equilibrium simulations. NgBamA requires the least energy to open its gate, explaining why it was observed in 2 μs. *E. coli* and *S. enterica* systems, on the other hand, require the most energy, consistent with no lateral gate opening observed in equilibrium simulations. Also, after 2-4 Å of separation, all the PMFs of gate opening in BamA and TamA are lower than the respective FhaC systems (with or without POTRA domains; Fig 3). Additional evidence for a functional role of lateral gating in BamA comes from a recent structure in which two BamAs, both with open gates, are in contact [37].

We also analyzed the thickness of membranes around the BamA/TamA barrels to evaluate the passive model of OMP insertion, in which a destabilized membrane region near BamA accelerates the process. Our results clearly show that all BamAs and TamA exhibit a narrowed hydrophobic region in their native outer membranes near the lateral gate. The consistency of this observation across multiple species and membrane compositions strongly supports a key tenet of the passive model.

Overall, our results provide evidence supporting both the hybrid barrel and passive models of BamA-mediated insertion of OMPs. However, these models do not necessarily contradict each other, but rather each may reflect an aspect of the insertion process. Our PMFs indicate that the lateral gate of BamA from some species may not open spontaneously, at least in the absence of some stimulating factor(s). This factor could be, for example, interaction with a nascent OMP, aided by the thinned membrane. Additionally, the positions of accessory proteins may alter the gate structure [17, 18, 20]. For example, BamB may stabilize the open lateral gate through its interactions with the POTRA domains. Although BamB is absent in the BAM complex of *N. gonorrhoeae*, our results demonstrate that its BamA is able to perform spontaneous lateral gate opening. Other accessory proteins, such as the essential BamD, may serve similar roles in regulating the conformation of BamA.

## Supporting information

S1 File Alignment of BamA, TamA and FhaC. (XLSX)

S1 Table Composition of systems built for this study. (PDF)

S2 Table Centers and force constants for replica-exchange umbrella sampling simulations. (PDF)

S3 Table Average surface areas of POTRA domains over time. (PDF)

S1 Fig Starting states for REUS. For BamA of *N. gonorrhoeae* and TamA of *E. coli*, we selected an open state observed in equilibrium simulations to use as a target in Targeted Molecular Dynamics (TMD) for generation of starting states for REUS. For other BamAs for which an open state was not observed in their own equilibrium simulations, we set the target to the backbone of the β-strands of the NgBamA target according to the alignment, and we use TamA as a target for FhaC (See S1 File. for the alignment).
(PNG)

S2 FigCoordinates for REUS.$\vec{D}$ is the direction that lateral gates open and $\vec{d}$ is the vector formed by N and O atoms that potentially form hydrogen bonds at the lateral gate. For example, the collective variable for BamA of *N. gonorrhoeae* is defined as the average of the distance between 432 N and 788 O and the distance between 432 O and 788 N projected to the vector between them in the target. N and O of Y432 and I806 are used for BamA of *E. coli*, Y432 and I800 for *S. enterica*, Y429 and V789 for *H. ducreyi*, W432 and L788 for *N. gonorrhoeae*, Y274 and I571 for TamA of *E. coli*, and N219 and I548 for FhaC of *B. pertussis*. The maximum separation and a close state are overlapped on the left with the maximum separation shown in blue and the close state in white. The atoms forming hydrogen bonds at the lateral gate are shown as spheres and colored by name on the right.(PNG)

S3 Fig PMFs of NgBamA system at different sampling intervals. We use 45-100 ns as the final sampling interval for NgBamA. For each umbrella sampling simulation, we calculated PMFs using different sampling intervals. When an additional 5-ns of sampling at the endpoint does not change the PMF by more than 0.2 kcal/mol, the simulation is considered converged.
(PNG)

S4 Fig Average membrane hydrophobic thickness map over 2-μs equilibrium simulations of BamA and TamA. (PNG)

S5 Fig The area of the thinned membrane where the thickness is below 20 Å. (PNG)

S6 Fig Average membrane thickness around the barrel for each system. (PNG)

S7 Fig Hydrophobic surface map of TamA. (PNG)

S8 Fig Strand separation and number of hydrogen bonds between the backbones of β1 and β16 in equilibrium simulations by time for BamA and TamA. (PNG)

S9 Fig PMF and number of hydrogen bonds formed between water and backbone of β1 and β16. (PNG)

S10 Fig Interaction propensities of each β-barrel with its native environment. For each residue, we add up the number of atoms of water, core, lipid A head group, lipid A tails, PL tails, PL head group, and protein (backbones of the nearby residues are excluded) within 4 Å of the side chain, respectively, over the 4 μs of combined equilibrium simulations. The graph shows the ratio of each category.
(PNG)

S11 Fig The method to measure the difference of interaction propensities. (PNG)

S12 Fig Water-exposure of extracellular loop residue side chains. For each residue, the fraction of surface area in contact with water is determined for each frame; the resulting list over the entire 2×2-μs trajectories is binned into 10 deciles and plotted. The darker the bar for a given residue, the more it is exposed to water.
(PNG)

S13 Fig Contact areas of POTRA domains with the periplasmic leaflet of the OMs. (PNG)
