# Supplementary material for: Membrane thinning and lateral gating are consistent features of BamA across multiple species
Source: PLoS Comput Biol. 2020 Oct 28;16(10):e1008355. doi: 10.1371/journal.pcbi.1008355 (PMC7652284; doi:10.1371/journal.pcbi.1008355)
Supplement: S1 Table — (PDF) [file pcbi.1008355.s002.pdf]

Label	Species	Protein		Membrane		Size	Atoms
		PDB	Residue	Outer Leaflet	Inner Leaflet		
EcBamA	*E. coli*	5D0O	24-810	106 LPS	226 PPPE, 60 PVPG, 15 PVCL2	150×150×164	371k
EcBamAcw	*E. coli*	5D0O	24-810	106 LPS	226 PPPE, 60 PVPG, 15 PVCL2	150×150×164	483k
EcBamAΔP	*E. coli*	5D0O	423-810	42 LPS	90 PPPE, 24 PVPG, 6 PVCL2	103×101×113	118k
SeBamAΔP	*S. enterica*	5OR1	423-804	43 LPS	105 PPPE, 15 PVPG, 5 PVCL2	106×99×122	126k
HdBamA	*H. ducreyi*	4K3C	262-793	41 LPS	9 DMPE, 85 PPPE,10 DPPG, 12 DYPG, 2 PMCL2, 2 TYCL2	99×99×161	139k
HdBamAΔP	*H. ducreyi*	4K3C	420-793	41 LPS	9 DMPE, 85 PPPE, 10 DPPG, 12 DYPG, 2 PMCL2, 2 TYCL2	99×99×139	118k
NgBamA	*N. gonorrhoeae*	4K3B	21-792	75 LPS	150 PPPE, 21 DPPG, 12 PYPG, 12 SOPG, 9 DPPC, 6 DYPC	128×128×176	254k
NgBamAΔP	*N. gonorrhoeae*	4K3B	420-792	47 LPS	100 PPPE, 14 DPPG, 8 DYPG, 8 SOPG, 6 DPPC, 6 DYPC	104×104×138	129k
EcTamA	*E. coli*	4C00	25-577	56 LPS	120 PPPE, 32 PVPG, 8 PVCL2	111×111×174	194k
EcTamAΔP	*E. coli*	4C00	265-577	42 LPS	90 PPPE, 24 PVPG, 6 PVCL2	99×99×135	114k
BpFhaC	*B. pertussis*	4QL0, 4QKY	1-554	48 LPS	46 DPPE, 5 DSPE, 24 DPPG, 10 DSPG, 16 DYPG, 23 PMCL2	108×106×155	179k
BpFhaCΔP	*B. pertussis*	4QL0, 4QKY	1-36, 210-554	40 LPS	39 DPPE, 4 DSPE, 21 DPPG, 8 DSPG, 14 PYPG, 20 PMCL2	99×99×143	120k
