# Supplementary material for: Membrane thinning and lateral gating are consistent features of BamA across multiple species
Source: PLoS Comput Biol. 2020 Oct 28;16(10):e1008355. doi: 10.1371/journal.pcbi.1008355 (PMC7652284; doi:10.1371/journal.pcbi.1008355)
Supplement: S2 Table — (PDF) [file pcbi.1008355.s003.pdf]

Centers (left) and Force Constants (right)										
EcBamA	1.70 5.0	2.50 5.0	2.85 12.5	3.25 17.0	3.5 25.0	3.80 22.5	4.10 21.0	4.40 17.0	4.70 15.0	5.05 12.5
	5.40 12.0	6.00 5.5	6.75 3.7	7.50 3.0	8.25 2.5	9.00 2.5	9.75 2.5	10.50 2.5		
EcBamAΔP	1.70 5.0	2.50 5.0	2.85 12.5	3.25 17.0	3.5 25.0	3.79 22.5	4.05 20.0	4.40 17.5	4.70 15.0	5.05 12.5
	5.40 12.0	6.00 5.5	6.75 3.3	7.50 3.0	8.25 2.5	9.00 2.5	9.75 2.5	10.50 2.5		
SeBamAΔP	1.70 5.0	2.50 5.0	2.85 12.5	3.25 17.0	3.5 25.0	3.70 22.0	3.95 18.5	4.30 10.0	4.70 6.5	5.20 4.7
	5.94 4.6	6.72 3.1	7.45 2.8	8.20 2.6	8.95 2.4	9.70 2.4	10.45 2.4			
HdBamA	1.70 5.0	2.50 5.0	2.85 12.5	3.25 17.0	3.5 25.0	3.79 22.0	4.00 17.5	4.50 7.5	5.20 5.5	6.00 5.0
	6.75 4.7	7.50 2.8	8.25 2.5	9.00 2.5	9.75 2.5	10.50 2.5	11.25 2.2			
HdBamAΔP	1.70 5.0	2.50 5.0	2.85 12.5	3.25 17.0	3.5 25.0	3.79 22.0	4.00 17.5	4.50 7.5	5.20 4.5	6.00 4.5
	6.75 3.3	7.50 3.0	8.25 2.5	9.00 2.5	9.75 2.5	10.50 2.5	11.25 2.2			
NgBamA	1.70 5.0	2.50 5.0	2.85 12.5	3.25 17.0	3.5 25.0	3.79 17.5	4.05 17.5	4.50 7.5	5.20 4.2	6.00 4.0
	6.75 3.5	7.50 3.0	8.25 2.5	9.00 2.5	9.75 2.5	10.50 2.5	11.25 2.2			
NgBamAΔP	1.70 5.0	2.50 5.0	2.85 12.5	3.25 17.0	3.5 25.0	3.79 17.5	4.05 17.5	4.50 7.5	5.20 4.2	6.00 4.0
	6.75 3.5	7.50 3.0	8.25 2.5	9.00 2.5	9.75 2.5	10.50 2.5	11.25 2.2	12.00 2.0		
EcTamA	3.00 18.5	3.30 25.0	3.60 25.0	3.90 22.0	4.30 17.5	4.70 12.0	5.20 11.0	5.70 10.0	6.10 9.0	6.50 8.5
	6.90 8.0	7.40 6.8	7.90 6.2	8.40 5.5	8.90 5.0	9.60 3.5				
EcTamAΔP	3.00 18.5	3.30 27.0	3.60 26.5	3.85 25.0	4.12 22.0	4.35 21.5	4.70 12.8	5.15 11.0	5.65 10.0	6.07 9.0
	6.50 8.5	6.90 8.0	7.40 6.8	7.90 6.2	8.40 5.5	8.90 5.0	9.60 3.5			
FhaC	2.50 15.0	3.00 18.5	3.30 25.0	3.60 27.0	3.90 26.0	4.20 21.0	4.50 18.0	4.80 15.0	5.10 11.0	5.50 9.0
	5.90 9.0	6.40 8.5	6.90 8.0	7.40 6.8	7.90 6.2	8.40 5.5	8.90 5.0	9.60 3.5	10.30 3.1	
FhaCΔP	2.40 15.0	2.80 18.0	3.10 20.0	3.34 25.0	3.60 27.0	3.90 26.0	4.20 21.0	4.50 19.0	4.80 17.0	5.10 15.5
	5.50 9.0	5.90 9.0	6.40 8.5	6.90 8.0	7.40 6.8	7.90 6.2	8.40 5.5	8.90 5.0	9.60 3.5	10.30 3.1
