# Supplementary material for: Membrane thinning and lateral gating are consistent features of BamA across multiple species
Source: PLoS Comput Biol. 2020 Oct 28;16(10):e1008355. doi: 10.1371/journal.pcbi.1008355 (PMC7652284; doi:10.1371/journal.pcbi.1008355)
Supplement: S3 Table — (PDF) [file pcbi.1008355.s004.pdf]

	BamA of *E. coli*	BamA of *N. gonorrhoeae*	BamA of *H. ducreyi*	TamA of *E. coli*
P5	5448.545 Å^2	5448.545 Å^2	5449.631 Å^2	
P4	5795.842 Å^2	5795.842 Å^2	6231.144 Å^2	
P3	6555.692 Å^2	6555.692 Å^2		5649.239 Å^2
P2	5407.943 Å^2	5407.943 Å^2		5656.914 Å^2
P1	4791.333 Å^2	4791.333 Å^2		6028.116 Å^2
