# Supplementary figures and images for: Membrane thinning and lateral gating are consistent features of BamA across multiple species
Source: PLoS Comput Biol. 2020 Oct 28;16(10):e1008355. doi: 10.1371/journal.pcbi.1008355 (PMC7652284; doi:10.1371/journal.pcbi.1008355)

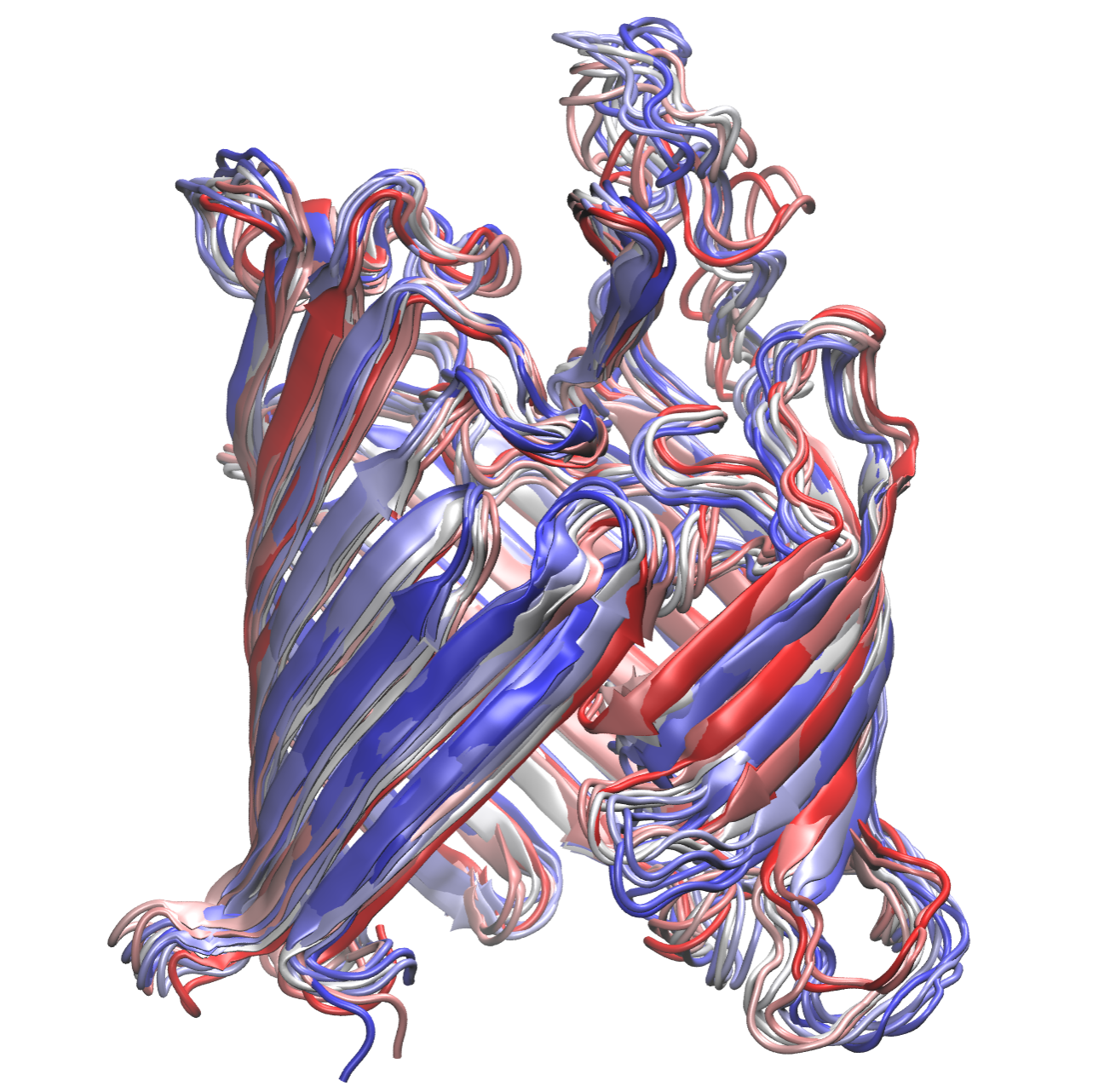

Supplement: S1 Fig — For BamA of N. gonorrhoeae and TamA of E. coli, we selected an open state observed in equilibrium simulations to use as a target in Targeted Molecular Dynamics (TMD) for generation of starting states for REUS. For other BamAs for which an open state was not observed in their own equilibrium simulations, we set the target to the backbone of the β-strands of the NgBamA target according to the alignment, and we use TamA as a target for FhaC (See S1 File. for the alignment). (PNG) [file pcbi.1008355.s005.png]

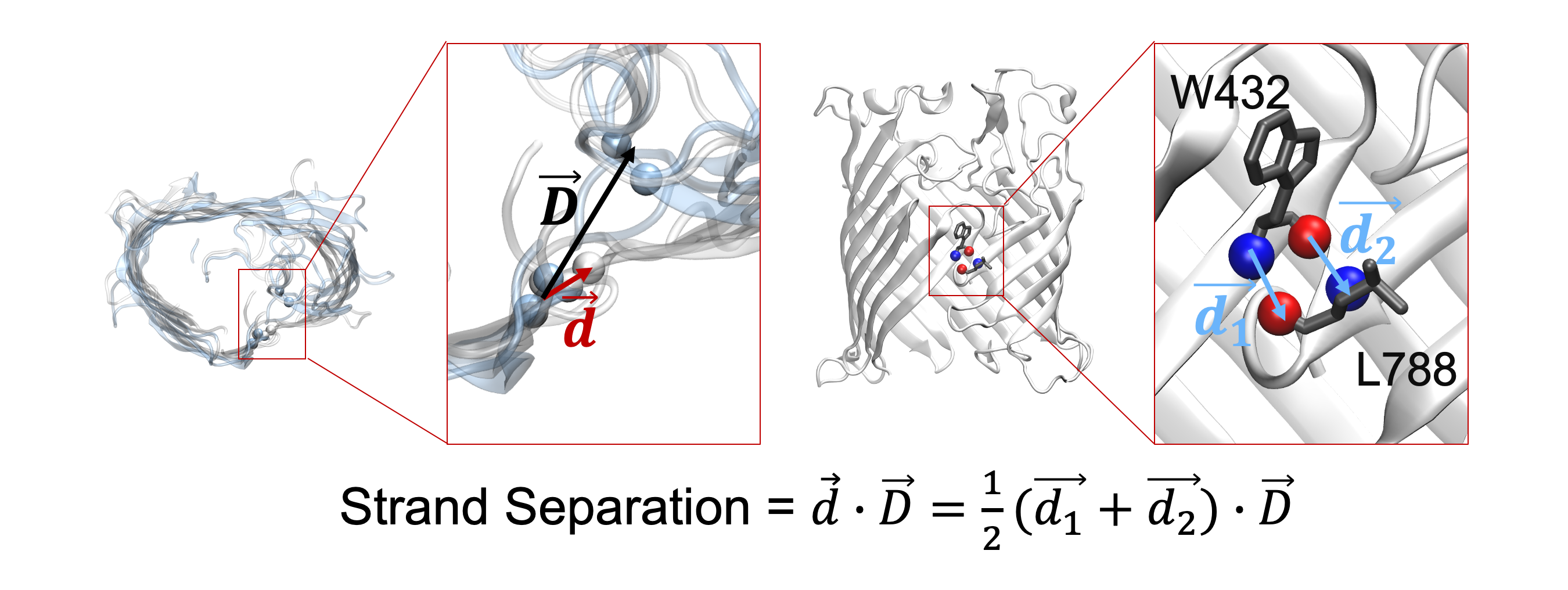

Supplement: S2 Fig — D→ is the direction that lateral gates open and d→ is the vector formed by N and O atoms that potentially form hydrogen bonds at the lateral gate. For example, the collective variable for BamA of N. gonorrhoeae is defined as the average of the distance between 432 N and 788 O and the distance between 432 O and 788 N projected to the vector between them in the target. N and O of Y432 and I806 are used for BamA of E. coli, Y432 and I800 for S. enterica, Y429 and V789 for H. ducreyi, W432 and L788 for N. gonorrhoeae, Y274 and I571 for TamA of E. coli, and N219 and I548 for FhaC of B. pertussis. The maximum separation and a close state are overlapped on the left with the maximum separation shown in blue and the close state in white. The atoms forming hydrogen bonds at the lateral gate are shown as spheres and colored by name on the right. (PNG) [file pcbi.1008355.s006.png]

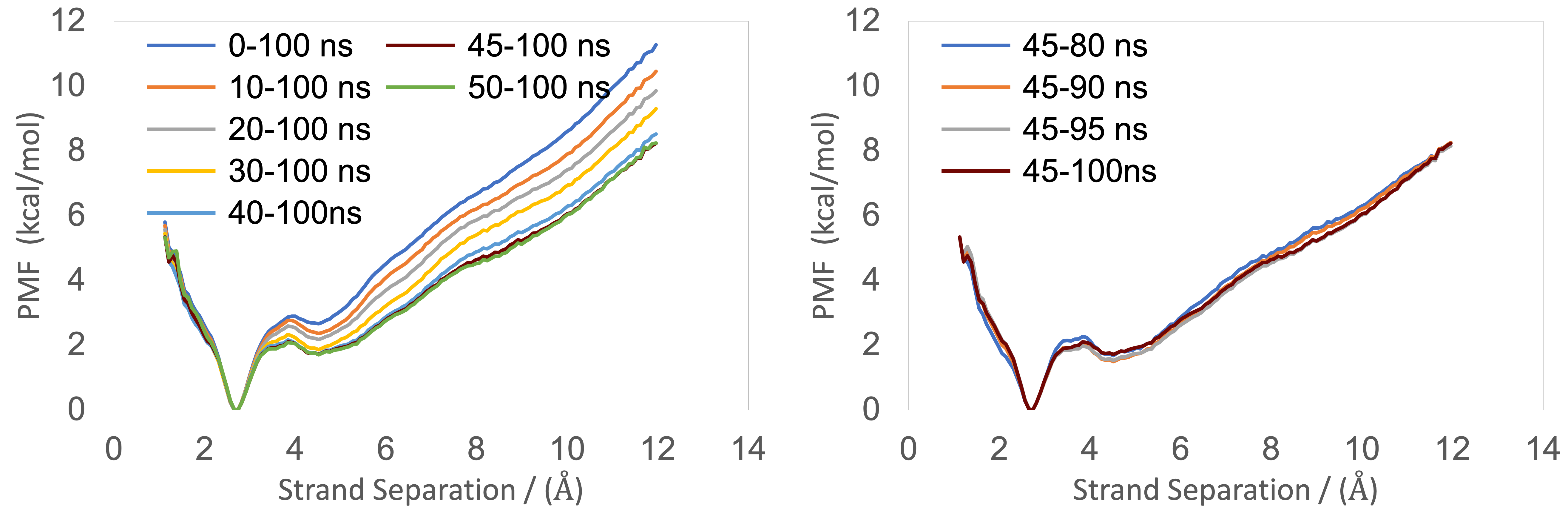

Supplement: S3 Fig — We use 45-100 ns as the final sampling interval for NgBamA. For each umbrella sampling simulation, we calculated PMFs using different sampling intervals. When an additional 5-ns of sampling at the endpoint does not change the PMF by more than 0.2 kcal/mol, the simulation is considered converged. (PNG) [file pcbi.1008355.s007.png]

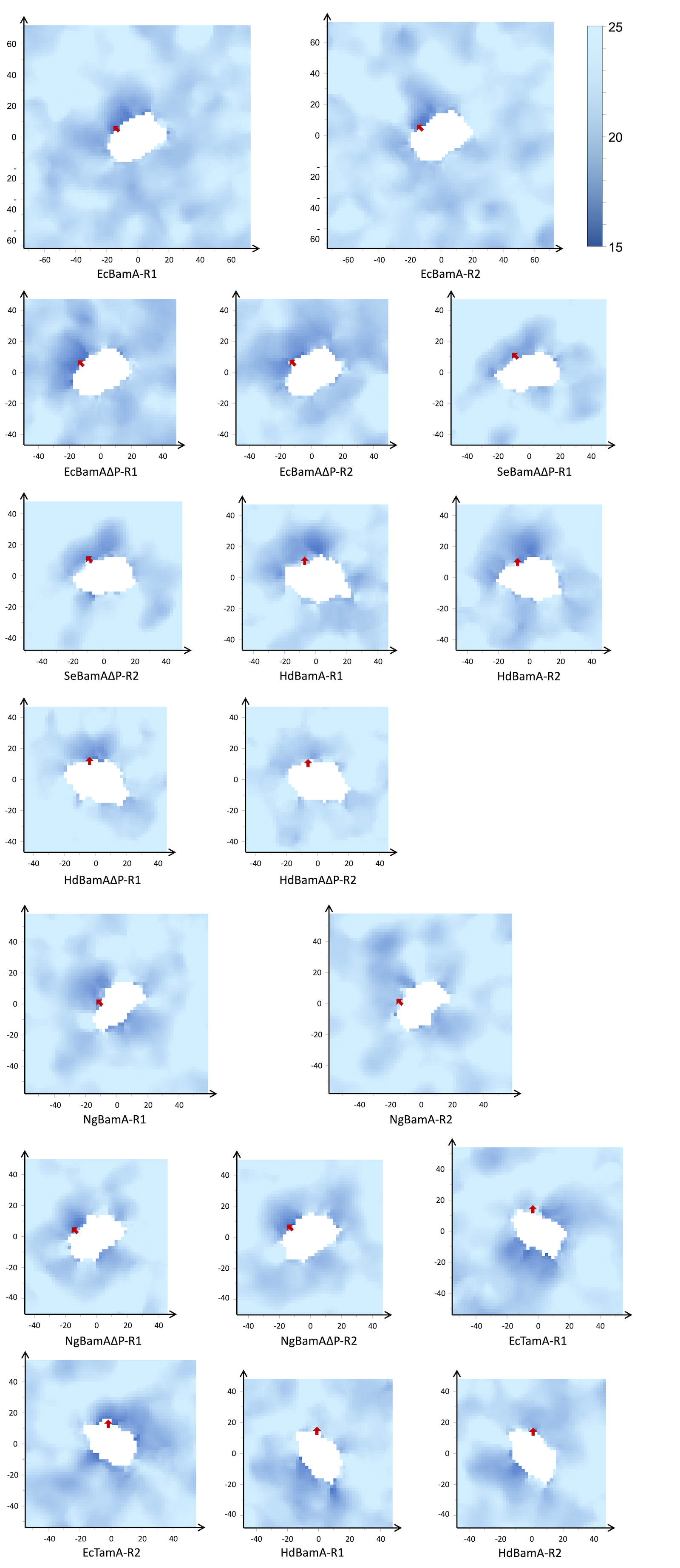

Supplement: S4 Fig — (PNG) [file pcbi.1008355.s008.png]

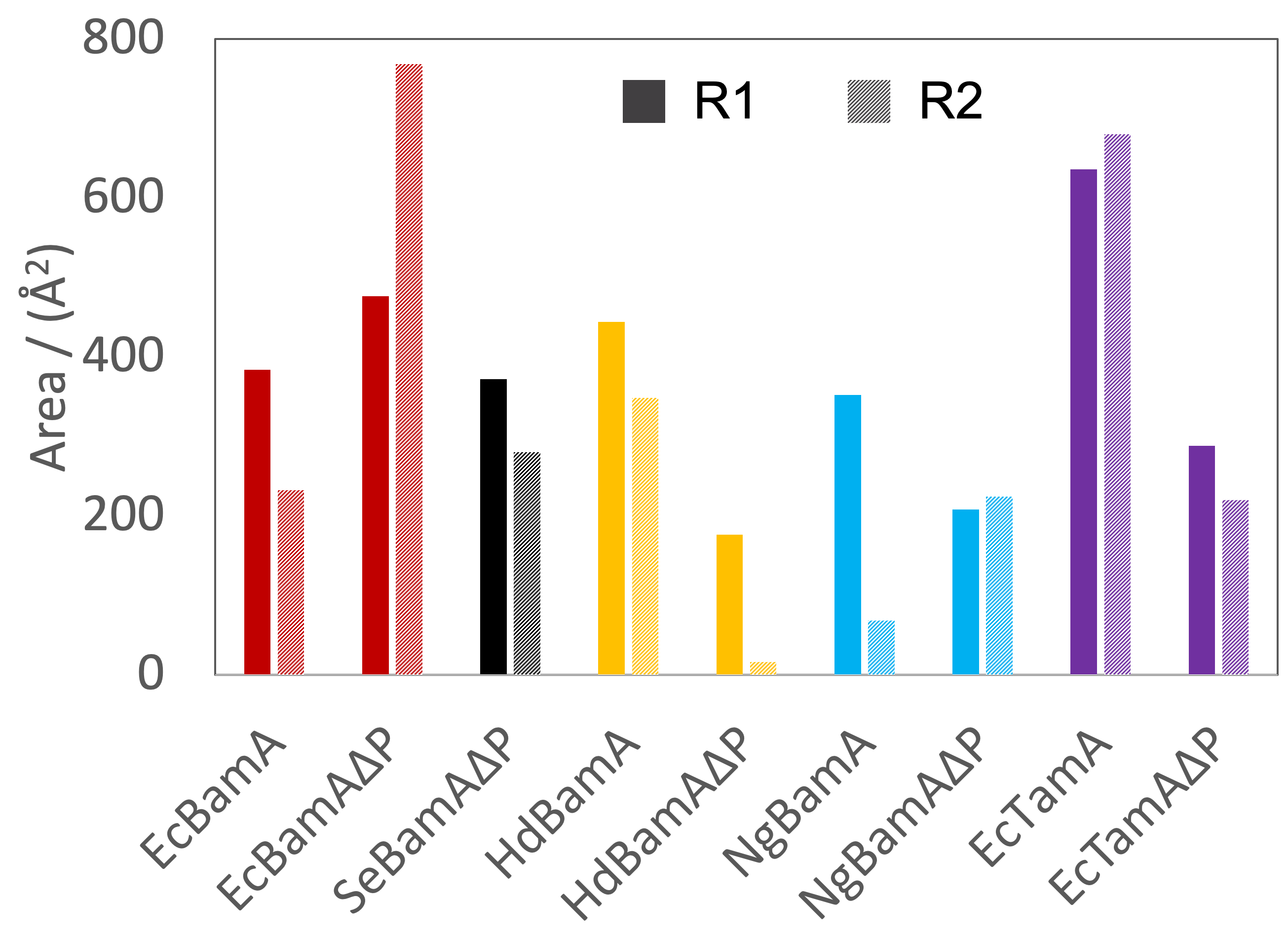

Supplement: S5 Fig — (PNG) [file pcbi.1008355.s009.png]

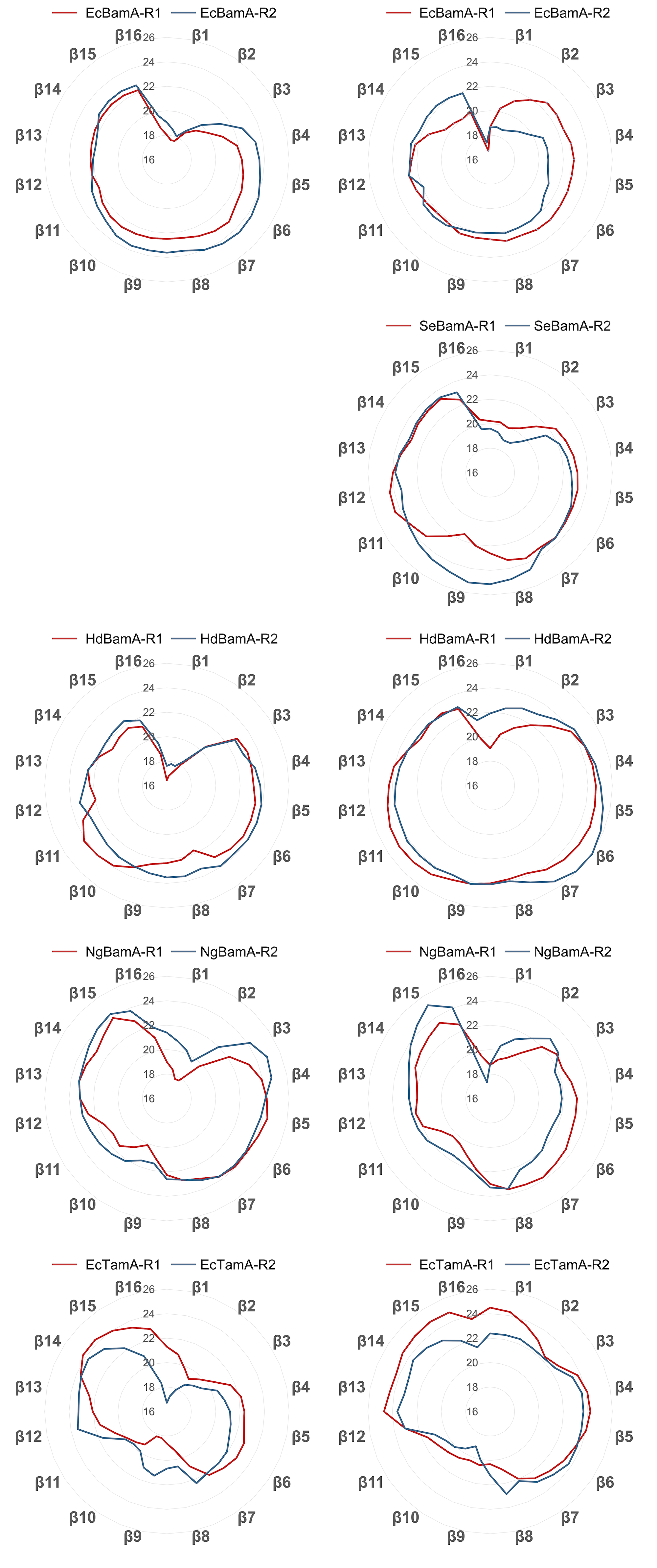

Supplement: S6 Fig — (PNG) [file pcbi.1008355.s010.png]

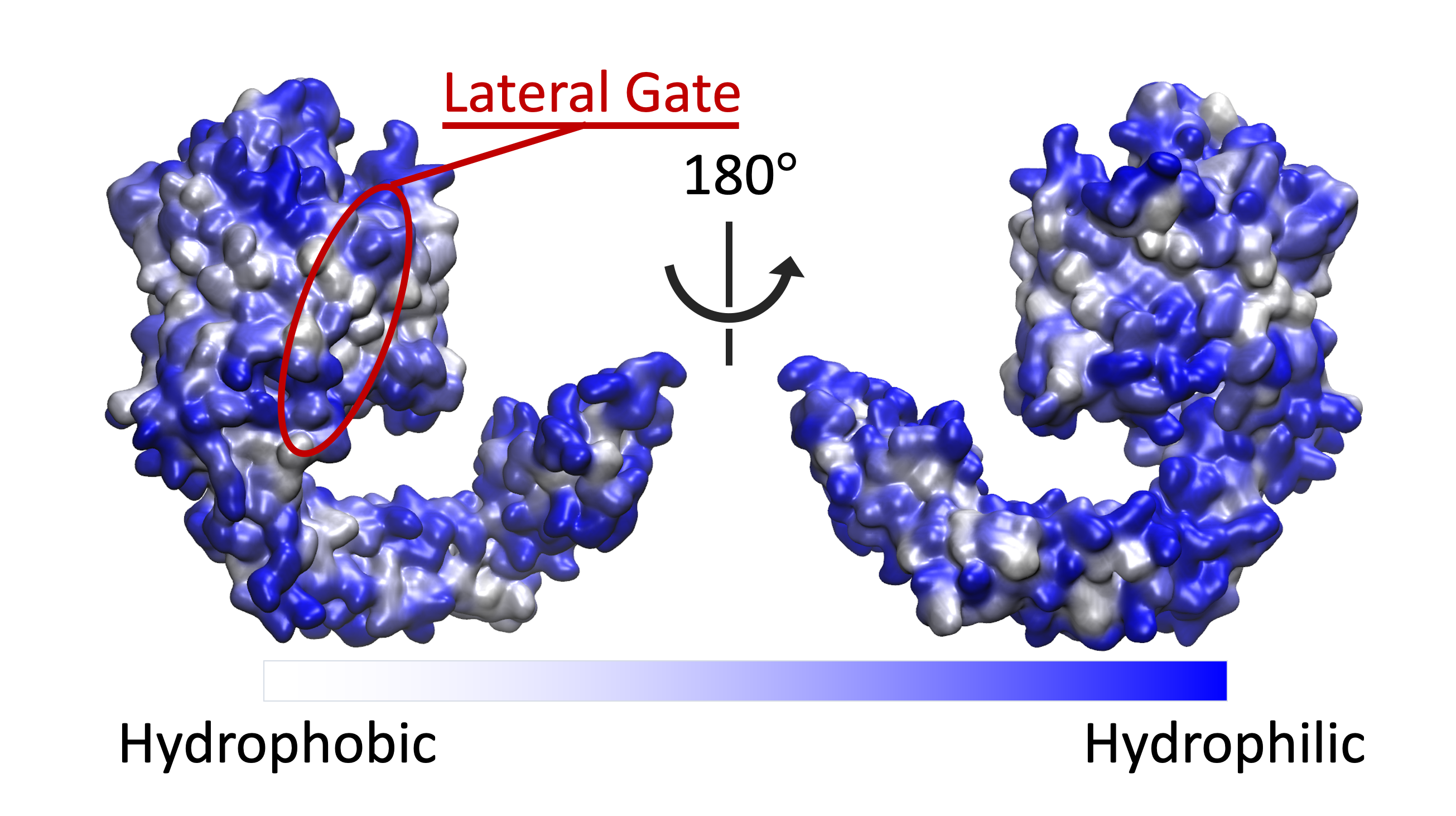

Supplement: S7 Fig — (PNG) [file pcbi.1008355.s011.png]

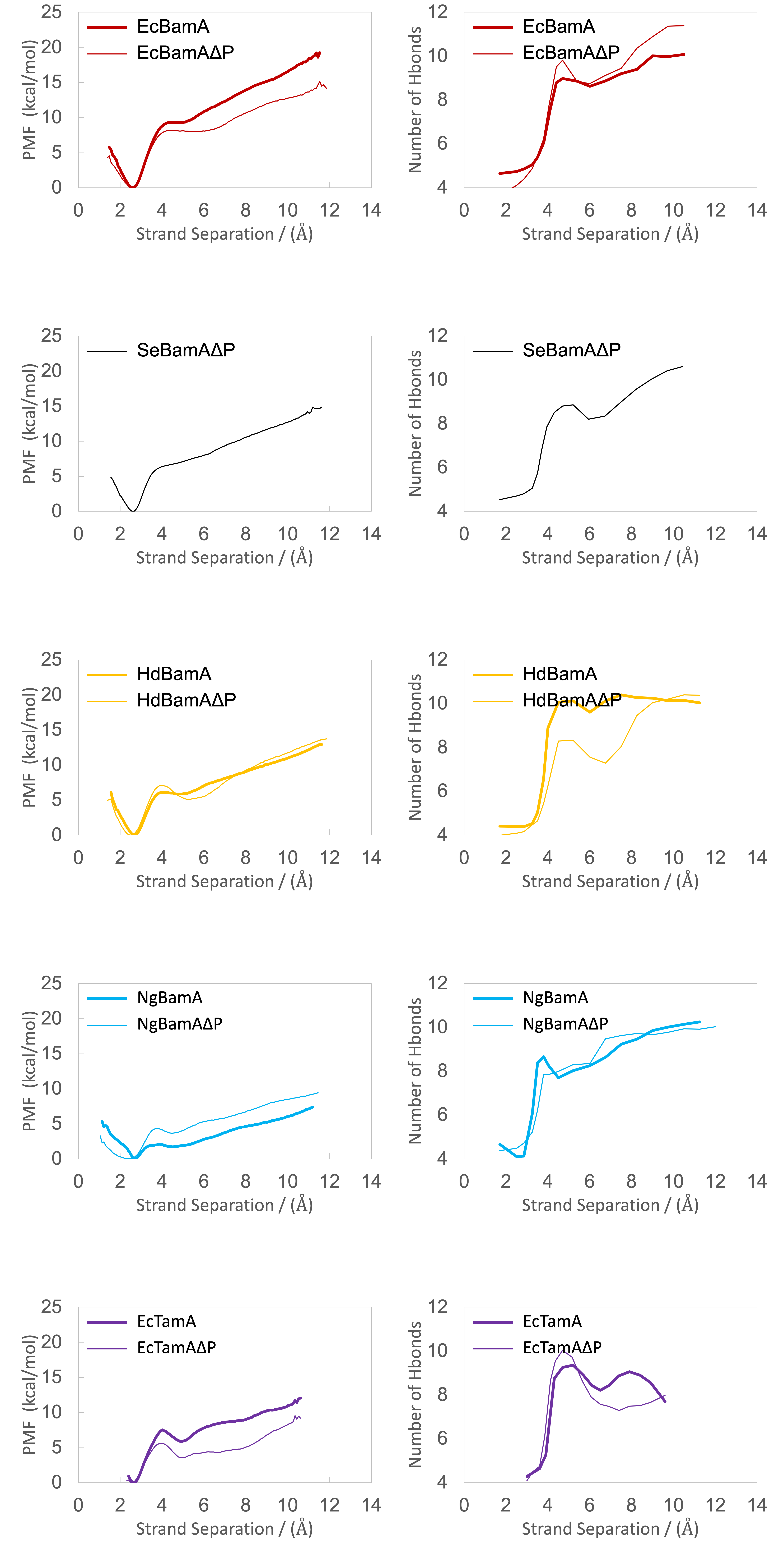

Supplement: S9 Fig — (PNG) [file pcbi.1008355.s013.png]

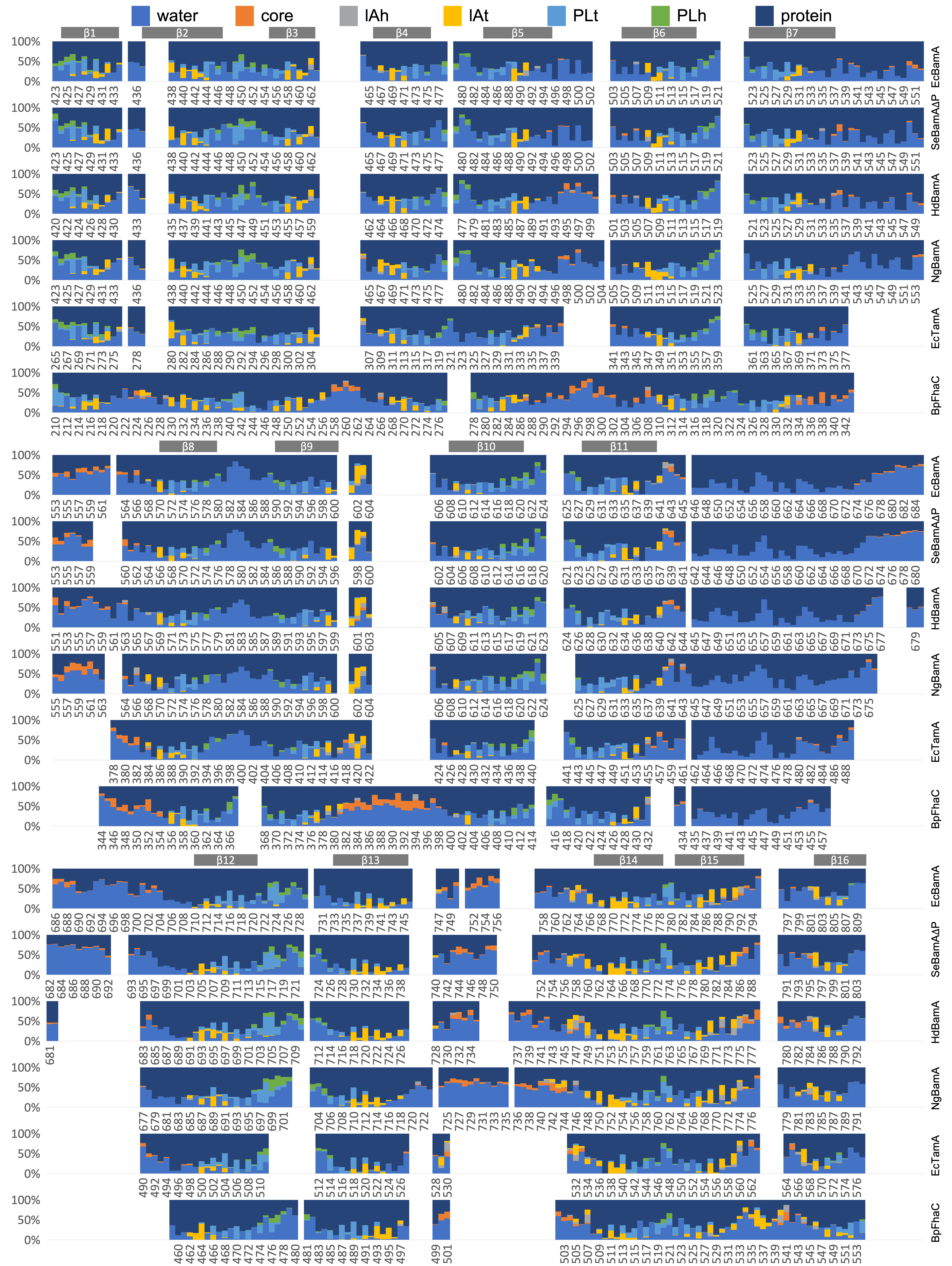

Supplement: S10 Fig — For each residue, we add up the number of atoms of water, core, lipid A head group, lipid A tails, PL tails, PL head group, and protein (backbones of the nearby residues are excluded) within 4 Å of the side chain, respectively, over the 4 μs of combined equilibrium simulations. The graph shows the ratio of each category. (PNG) [file pcbi.1008355.s014.png]

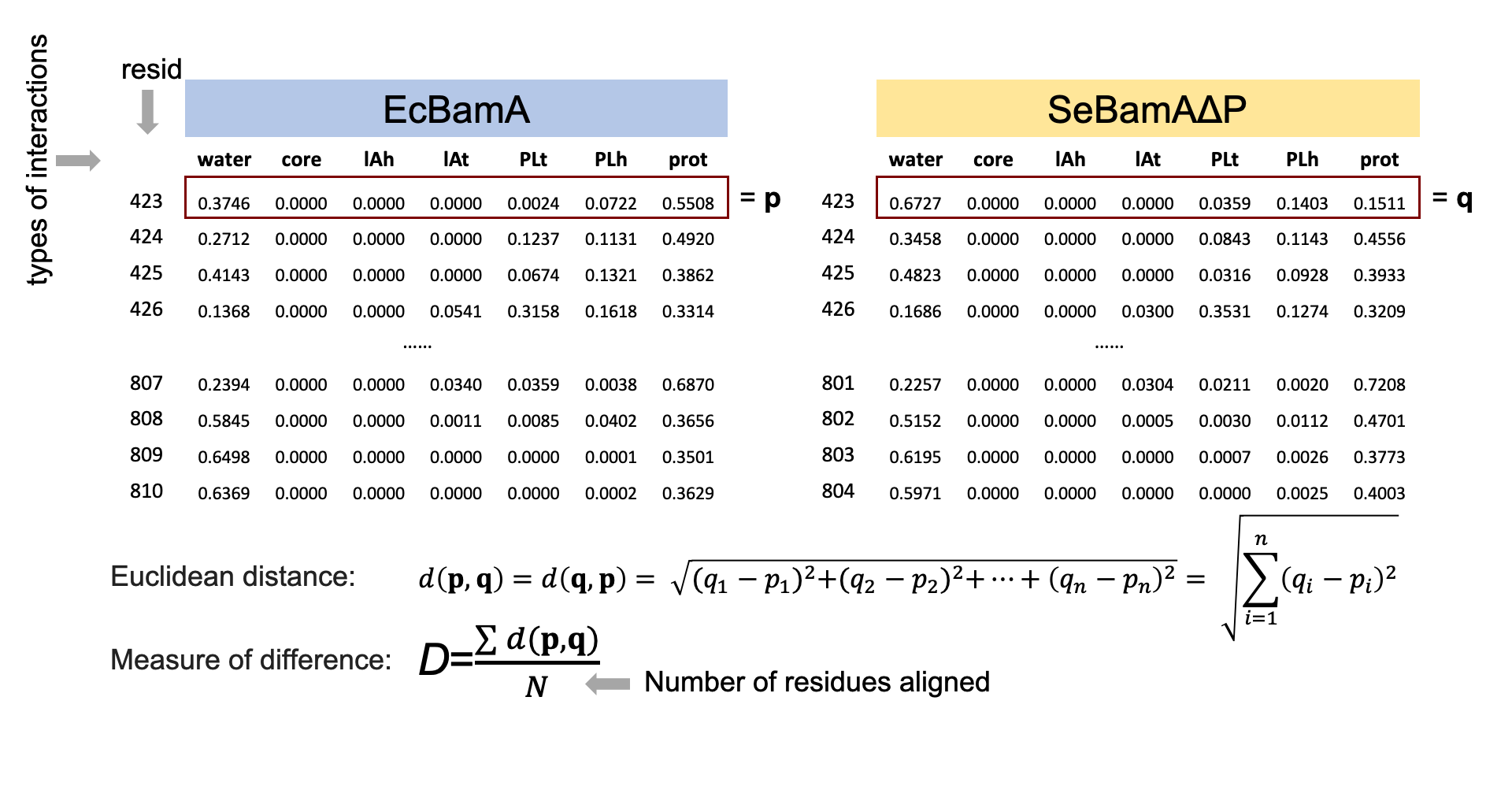

Supplement: S11 Fig — (PNG) [file pcbi.1008355.s015.png]

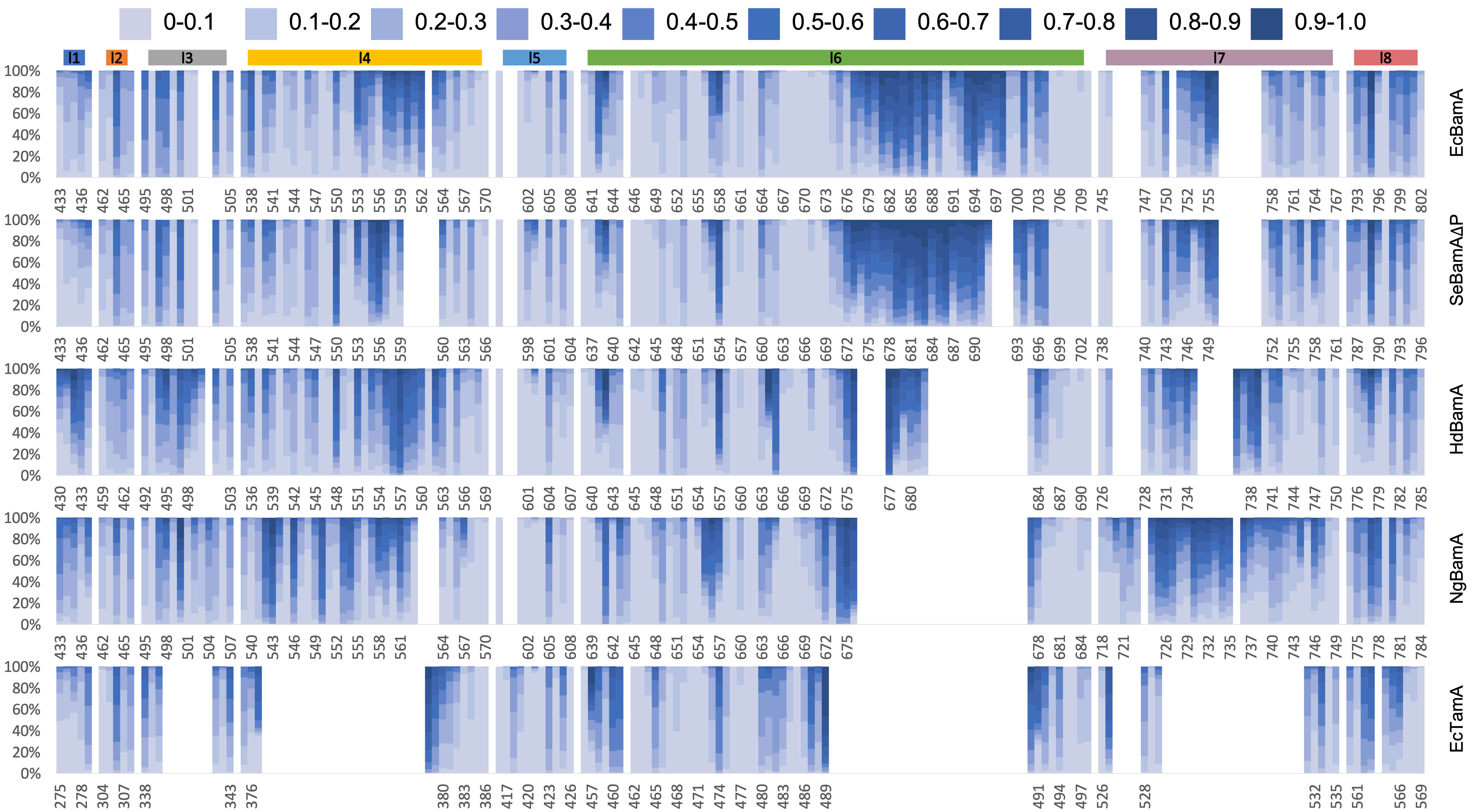

Supplement: S12 Fig — For each residue, the fraction of surface area in contact with water is determined for each frame; the resulting list over the entire 2×2-μs trajectories is binned into 10 deciles and plotted. The darker the bar for a given residue, the more it is exposed to water. (PNG) [file pcbi.1008355.s016.png]

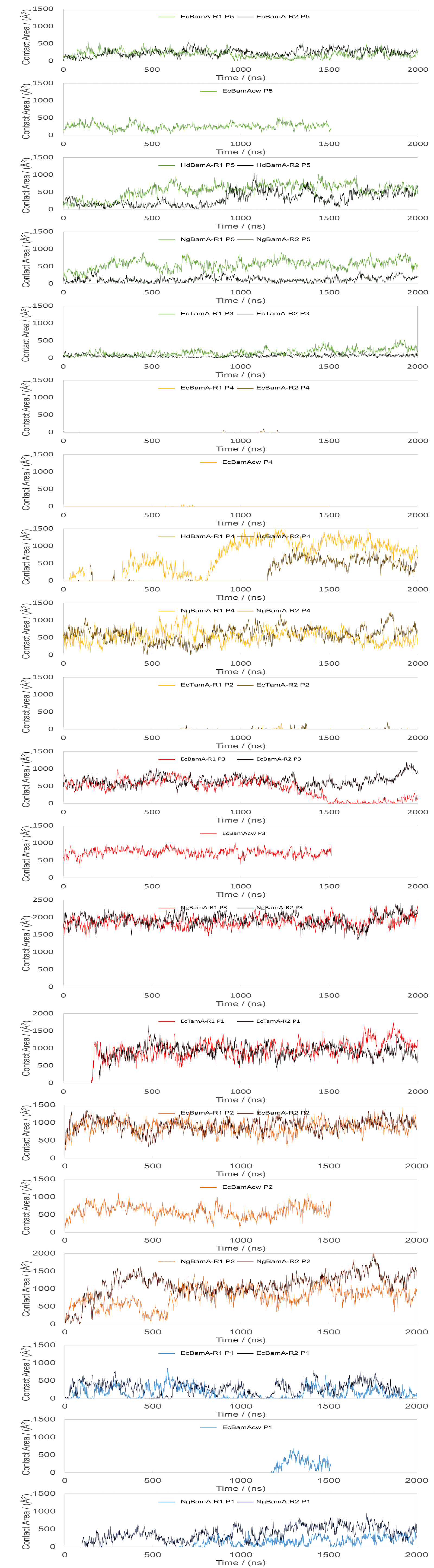

Supplement: S13 Fig — (PNG) [file pcbi.1008355.s017.png]
